# Outdoor thermal comfort and cognition impact pro-environmental behaviors: evidence from a field experiment in the tropics

**DOI:** 10.3389/fpsyg.2025.1472852

**Published:** 2025-05-05

**Authors:** Natalia Borzino, Samuel Chng, Renate Schubert

**Affiliations:** ^1^Singapore-ETH Centre (SEC), CREATE Tower, Singapore, Singapore; ^2^Campus for Research Excellence and Technological Enterprise (CREATE), CREATE Tower, Singapore, Singapore; ^3^Lee Kuan Yew Centre for Innovative Cities, Singapore University of Technology and Design (SUTD), Singapore, Singapore; ^4^Department of Humanities, Social and Political Sciences, Chair of Economics, ETH-Zurich, Zürich, Switzerland

**Keywords:** cognitive control capacity, pro-environmental behaviors, heat and climate change, outdoor thermal comfort (OTC), attitudes-behavior gap

## Abstract

**Introduction:**

Climate change and the Urban Heat Island (UHI) effect pose a serious threat, particularly for tropical countries like Singapore, which experience high air temperatures and humidity levels and are heating up twice as fast as the global average. Policy interventions have focused on promoting individuals' engagement in pro-environmental behaviors to mitigate urban heat and CO_2_ emissions. Although past research highlights individuals' long-lasting environmental attitudes and awareness, these do not always translate into action. This study investigates the *attitudes-behavior gap* and the *awareness-behavior gap* from a cognitive perspective, and examines the extent to which cognition is affected by urban heat.

**Methods:**

Using a quasi-experimental field design involving 309 older adults and a novel analytical framework, we assessed the relationship between thermal comfort, cognitive control, and pro-environmental behavior.

**Results:**

We found that low thermal comfort negatively affects cognitive control, which in turn significantly moderates the relationship between pro-environmental attitudes and behaviors, as well as between awareness and behaviors. Specifically, individuals with higher cognitive control capacity demonstrated a stronger moderating effect, helping to close the *attitudes-behavior* and *awareness-behavior gaps* and encouraging more pro-environmental behavior.

**Discussion:**

Policies aimed at preserving thermal comfort and enhancing heat adaptation can support not only the health and wellbeing of senior citizens but also their pro-environmental behaviors. This presents a potentially central lever for behavioral change initiatives.

## 1 Introduction

### 1.1 The urgency of climate action in response to climate change

It is unequivocal that human influence has led to climate change. The scientific community has been sounding the alarm and warning that urgent climate action is necessary (IPCC, 2023; United Nations Climate Change, 2024; USGCRP, 2023). They have emphasized that we can expect more extreme weather patterns with climate change. The Intergovernmental Panel on Climate Change (IPCC), in its report released in March 2023, projects that global surface temperature will continue to increase until at least the mid-century under all emissions scenarios considered. Independently, the Fifth National Climate Assessment (NCA5) released in November 2023 by the United States Global Change Research Program (USGCRP, 2023) reported the number and strength of heat waves, heavy downpours, and major hurricanes events in the United States alone have increased. Therefore, in the 29th session of the Conference of the Parties to the United Nations Framework Convention on Climate Change (United Nations Climate Change, 2024) held in November 2024, it was conveyed that the impacts of climate change are already being felt across the globe, disproportionately affecting the most vulnerable population groups and highlighted the need for increasing robust climate adaptation measures. This can be seen as a strong call to action on all fronts, including individuals, to play active roles in creating a more climate neutral world.

While governments and organizations are implementing actions to mitigate the effects of climate change and to create more sustainable economies and businesses, individuals can contribute through their everyday behavior. Indeed, household consumption is estimated to be responsible for 65% of global greenhouse gas emissions (Ivanova et al., 2016). In this sense, the United Nation's Sustainable Development Goal 12 (“Sustainable Consumption and Production”; UN DESA, 2017) demonstrates a large consensus that today's consumption patterns are unsustainable and changes in consumer behaviors are urgently needed (Ivanova et al., 2020).

However, making individuals adopt more pro-environmental actions is challenging (Spangenberg and Lorek, 2019), as socio-economic and cultural systems (Vesely et al., 2022; Pong and Tam, 2023), emotions (Panno et al., 2020; Carrus et al., 2021; Nielsen et al., 2024), social and psychological factors (e.g., environmental attitudes and awareness) and cognitive factors matter (Zwicker et al., 2020; Linder et al., 2022; Shen and Wang, 2022; Kühn and Bobeth, 2022). Therefore, it is important to explore the role of these factors, as well as their interrelationship, in igniting (or hindering) sustainable behaviors (Muñoz, 2017; Jaiswal and Singh, 2018; Jaiswal et al., 2021).

### 1.2 Bridging the gap between pro-environmental attitudes, awareness and behaviors

Past environmental research highlighted the need to better understand the cognitive processes underlying pro-environmental behaviors to develop more effective climate action interventions and policies (Clayton et al., 2015; Bamberg, 2013; Nielsen, 2017). This could be also an explanation of why pro-environmental attitudes are not always translated into pro-environmental behaviors, referred to as the environmental *attitudes-behavior gap* (Juvan and Dolnicar, 2014; Kennedy et al., 2009; Nielsen, 2017). Past studies have explored whether, to what extent and under which conditions behavioral interventions aimed to increase the level of attitudes could effectively increase pro-environmental behavior. Evidence shows the low effect of intervention studies and information campaigns targeted at attitudes on actual behavior in the health domain (see meta-analysis; Michie et al., 2009) and in household natural resources consumption (Abrahamse et al., 2005). Instead, behavioral studies that put emphasis on making pro-environmental behaviors less cognitively effortless have reported it being more effective in shaping decision making and resulting in a more persistent effects over time (Borzino et al., 2025; Ebeling and Lotz, 2015; Tiefenbeck et al., 2016). This highlights the importance of focusing on cognition as a bridge between attitudes and behaviors.

When evaluating the pro-environmental behaviors, the role of beliefs (or misbelief) about climate change must not be overlooked. Past studies have found that sustainability- and environment-related awareness is also related to pro-environmental behaviors (Berger and Wyss, 2021; Osbaldiston and Schott, 2012). However, pro-environmental awareness does not always seem to translate into actions (Osbaldiston and Schott, 2012). This phenomenon is referred to as the *awareness-behavior gap*. In their meta-analysis, Osbaldiston and Schott (2012) found that interventions with cognitive elements were the most effective in increasing awareness and shaping pro-environmental behaviors. These cognitive elements included strategies like cognitive dissonance, setting goals, using prompts, and social modeling. In the decade since their review, there have been an increasing number of experimental studies focusing on exploring in depth diverse cognitive elements to increase awareness of different environment-related challenges (e.g., Al-Marri et al., 2018; Berger and Wyss, 2021; Cogut et al., 2019). The experimental evidence suggests the importance of cognition as a way to close the gap between awareness and behaviors.

### 1.3 Cognitive control capacity and its role in influencing pro-environmental behavior

Given the role of cognition, environmental researchers theorize that cognitive control capacity could be an important determinant of sustainable behaviors (Nielsen, 2017; Weber, 2017). Considering that pro-environmental behaviors are not intuitive behaviors but require individuals to be aware, deliberate and intentional, individuals' cognitive control capacity seems to matter. Cognitive control capacity is broadly defined as a set of mental processes that guide the intentional selection of behaviors for specific tasks while engaging in concomitant suppression of inappropriate and competing alternative actions (Redick, 2014; Miller and Cohen, 2001). In short, cognitive control is the process by which goals or plans influence behavior. It allows to deliberately inhibit a dominant, automatic or prepotent response (e.g., eating a piece of cake) to maximize the individuals' best interests (e.g., lose weight or stay healthy). In this sense, it is relevant to explore whether and how cognitive control capacity influences the translation of pro-environmental attitudes and awareness into pro-environmental behaviors, hereby helping to close both the *attitudes-behavior gap* and the *awareness-behavior gap*.

In a recent experimental study, Langenbach et al. (2020) investigated the role of cognitive control capacity. They focused on the attitude-behavior gap of young university students and found that participants' cognitive resources, specifically their cognitive control capacity, supported the translation of pro-environmental attitudes into a broad set of everyday pro-environmental behaviors (e.g., recycling). Participants with higher cognitive control capacity presented a behavior that was closely related to their attitudes. This finding is important for the endeavor to close the attitude-behavior gap. Yet, more research is needed to study whether this relationship applies to a broad range of pro-environmental behaviors, settings and socio-economic demographics other than university students.

### 1.4 Impact of heat on cognitive control capacity and pro-environmental behavior: our hypotheses

Given the importance of cognitive control capacity, it is also crucial to explore which external conditions could impact it as well as derived ways to preserve, enhance or encourage pro-environmental behaviors if pro-environmental attitudes and awareness are given. Existing studies suggest that cognitive control capacities could be affected by the environmental conditions to which individuals are exposed, for example heat. Past research suggests that heat exposure might affect cognitive control performance (e.g., Russell et al., 2020; Chea et al., 2025; Anderson, 1989; Anderson et al., 1997; Taylor et al., 2016; Gaoua et al., 2018; Laurent et al., 2018; Zhang et al., 2019; Lan et al., 2022). For example, heat stress is associated with aggressive behavior (e.g., Anderson, 1989; Anderson et al., 1997), which at the same time is associated with impulsivity and low cognitive control (Nakata et al., 2021; Meidenbauer et al., 2025; Chang et al., 2017). Other evidence suggests high temperature might affect cognitive control through the impairment of tasks performance following heat stress, decrease attention span (with more attention demanding tasks being more vulnerable than less attention demanding tasks) and information processing (for a review, see Hancock and Vasmatzidis, 2003; Schertz et al., 2022; Taylor et al., 2016; Yin et al., 2024; Malcolm et al., 2018).

Climate change is causing an increase in temperatures, which impact citizens' thermal comfort, particularly when it is combined with the effect of other environmental variables like relative humidity, wind speed or solar radiation (Vukmirovic et al., 2019). People living in tropical and subtropical countries often experience thermal comfort that is compromised by the rise of temperatures and humidity levels (Orosa et al., 2014) or due to the urban heat island effect coming along with the rapid growth of cities (Marcotullio et al., 2021). The urban heat island effect exacerbates climate change impacts by increasing temperatures in large tropical cities like Singapore to up to 7°C compared to rural areas (Roth and Chow, 2012).

**Figure 1:**
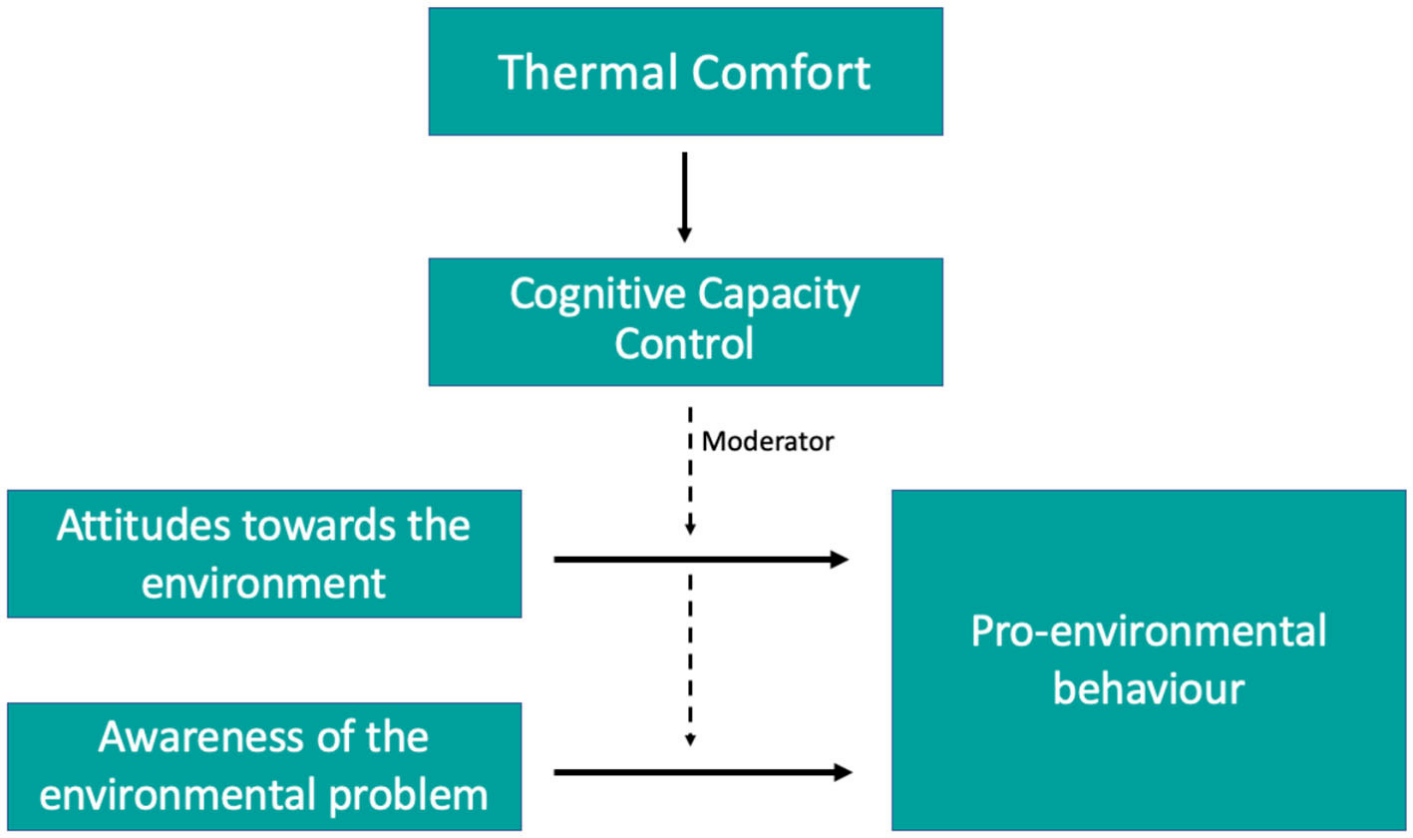
Relationship between environmental attitudes and awareness, cognitive control capacities and environmental behaviors.

Therefore, and given the evidence provided above, we can hypothesize that exposure to thermal conditions outside of comfort levels could weaken people's cognitive control capacities, which would attenuate citizens' pro-environmental behaviors. Figure 1 displays our presumptions in more detail. Low thermal comfort could impact cognitive control capacity, which at the same time moderates the relationship between environmental attitudes as well as awareness on the one side and environment-related behaviors on the other side, helping to close both the *attitudes-behavior gap* and the *awareness-behavior gap*.

Despite its relevance, there is only a small number of studies conducted in tropical countries analyzing the effect of heat on the cognitive resources of the young and adult population (e.g., Ndetto and Matzarakis, 2013; Porras-Salazar et al., 2018; Lipczynska et al., 2018) and of the elderly population (e.g., Wu et al., 2019; Hwang and Chen, 2010; Ma et al., 2021). Further insights on the impact of heat on the cognition of older adults will be increasingly important as the population living in tropical countries account for almost half the global population and many of the tropical countries have a rapidly aging population (United Nations, 2023).

### 1.5 Research aims and objectives

In this paper, and following Figure 1, we want to investigate whether (1) thermal comfort affects cognitive control capacities of healthy older adults living in tropical countries, more precisely in Singapore. If so, we want to recommend measures on how to strengthen the cognitive capacities of older adults in tropical countries; and (2) cognitive control capacity is a moderator in the gap between pro-environmental attitudes as well as awareness of an environmental problem and pro-environmental behaviors in older adults.

Our research builds on the existing literature on the relationship between thermal comfort and cognition of older adults (Wu et al., 2019; Hwang and Chen, 2010; Ma et al., 2021) as well as on the framework developed by Langenbach et al.'s (2020). However, our study presents some novel features. First, we conduct the study with participants in outdoor environments in Singapore where they carry out everyday activities. This helps to understand the potential impact of outdoor thermal conditions on cognitive control capacities in the course of daily living. Second, we focus on older adults, who are a vulnerable segment of countries' populations and who are of growing importance for aging societies. Third, we investigate the specific pro-environmental behavior of air conditioning use, which is a common lifestyle behavior in Singapore that has a considerable impact on energy consumption and contributes to urban heat effects. And lastly, to measure cognitive capacity, we focused on “selective attention capacity” because it declines with age. This variable is heavily influenced by extreme thermal conditions, and it seems of specific relevance for older adults.

The remainder of the paper is structured as follows: Section 2 describes the methods. Section 3 presents the results while Section 4 contains the concluding remarks and recommendations.

## 2 Materials and methods

### 2.1 Participants

We conducted a quasi-experiment in naturalistic outdoor environments in Singapore. Three hundred and nine healthy older adults were recruited in a total of 10 data collection sessions and on average each session lasted 80 min. Participants gave written informed consent before their participation. Older adults who were unable to provide their informed consent were excluded in compliance with ethical concerns. Ethic approval was obtained before the starting of the study. The average compensation for participating in the study was SG$20 per person plus an additional fixed amount of SG$5 for completing the survey questionnaire. Thus, participants could earn a total of SG$25 (around US$18).

### 2.2 Measures

In this section, and following our framework display in Figure 1, we describe the main measures used in our analysis to estimate the different variables of interest.

#### 2.2.1 Cognitive control capacity

We use a game based on the Stroop task, a seminal measure of cognitive control, to build an indicator for the cognitive control capacity. The Stroop task is widely used to measure the ability to inhibit cognitive interference. Previous literature also reports its application to measure other cognitive functions such as attention span, processing speed, cognitive flexibility (Jensen and Rohwer, 1966), or working memory (Kane and Engle, 2003). In the Stroop task, an individual is required to focus on task-relevant stimuli properties (i.e., identifying the colors of printed words), while holding back prepotent responses (i.e., reading the words regardless of the color). Hence, it explores the performance cost in a mismatch condition relative to a control condition (MacLeod, 2023; Scarpina and Tagini, 2017). In our study, the control condition tasks (congruent questions) were those, for which the ink color coincided with the color name flashed on a tablet screen. Mismatch condition tasks (incongruent questions) were given when the ink color was different from the color name. Congruent questions mimic easy tasks in real life that require lesser cognitive effort while incongruent questions simulate difficult tasks that require more cognitive effort and longer processing time (MacLeod, 2023). The Stroop (or interference) effect was calculated as the difference in time needed for each participant to answer congruent and incongruent questions. Participants were asked to perform the Stroop test twice during the session (see Section 2.3 for details). The cognitive control capacity was calculated by the averaged interference effect from the two Stroop tests performed by each subject.

An equal number of eighteen congruent and eighteen incongruent questions were presented in each test in randomized order. The participants were required to indicate their response to each question by selecting the answers presented at the bottom of the tablet screen within 5 s; after 5 s, the current question on the screen was replaced by the next one. There were three possible types of responses: correct, wrong or missed. As the Stroop task required participants to differentiate between colors and read basic color names, older adults who suffer from color blindness (assessed using the Ishihara color-blindness test) and/or those who were unable to read color names were excluded.

#### 2.2.2 Thermal comfort

To capture the level of outdoor thermal comfort, we used two alternative indexes: the Physiological Equivalent Temperature (PET) and Wet Bulb Globe Temperature (WBGT) (see Supplementary material for more details and equations). Both indexes are widely used in the literature and are considered as proxy for outdoor thermal comfort by capturing how changes in the thermal environment can affect an individual's outdoor thermal comfort (Deb and Ramachandraiah, 2010; Heng and Chow, 2019). By using both indexes in our analysis, we aimed to validate the consistency of our results, confirm their robustness and increase comparability with past and future evidence.

PET and WBGT present similarities (see Deb and Ramachandraiah, 2010): (1) they are measured in degrees Celsius and so they can be easily related to common experience; (2) they do not rely on subjective measures and (3) they are useful in both hot and colder climates. The interpretation of the both indexes is straightforward: the higher (lower) the PET and the WBGT indexes, the lower (higher) the outdoor thermal comfort.

We used mobile kestrels to capture the outdoor climatic conditions (i.e., air temperature, relative humidity, wind speed and the globe temperature) during the sessions. These environmental variables allowed us to calculate the PET and WBGT indices.

#### 2.2.3 Pro-environmental behaviors

Air-conditioning is one of the most important sources of energy consumption in humid and tropical countries like Singapore. It is also one of the major sources of anthropogenic heat and CO2 emissions. Hence, we consider resource conservation due to less air-conditioning usage as one of the most important pro-environmental behaviors. Therefore, the usage of air-conditioning is taken as a measure for everyday pro-environmental behavior of the participants. Specifically, we asked the participants to state how frequently they use the air-conditioning at home. The participants needed to choose one out of 5 options starting from “no usage” (1); “once every two weeks” (2); “one to two times a week” (3); “two to three times a week” (4); or “ everyday” (5). Accordingly, the pro-environmental behavior of each participant could range from 1 to 5.

#### 2.2.4 Attitudes toward the environment

The attitude toward the environment was taken as a measure for pro-environmental attitudes. The respective values were elicited by using the responses to the following two statements: “Mitigation action needs to be taken for Singapore's changing climate” and “More resources should be allocated to address the changes in climatic conditions faced in Singapore”. The responses were given on a 5-point Likert scale, ranging from “strongly disagree” (1) to “strongly agree” (5). We then computed one mean score for each of the participants, with scores ranging from 1 to 5, and with higher scores indicating a more positive attitude toward urban heat mitigation. The scales used to measure attitudes toward the environment demonstrated good internal consistency with Cronbach's Alphas equal to 0.85, with 0.7 typically seen as the lowest boundary for acceptance (Pallant, 2013).

#### 2.2.5 Awareness of environmental problems

The level of awareness of environmental problems related to climate change was taken as a measure for environmental awareness. It was measured by the responses to the three following statements: “The changing climate in Singapore is an urgent problem”, “Compared to 5 years ago, Singapore is much warmer now”, and “Compared to 5 years ago, Singapore is much cooler now”. The third statement was negatively worded as a consistency check. The responses were given on a 5-point Likert scale, ranging from “strongly disagree” (1) to “strongly agree” (5). The mean score was then computed per person and can range from 1 to 5, with 1 being the lowest level of climate change awareness and 5 being the highest level of climate change awareness. The scales used to measure awareness of an environmental problem demonstrated good internal consistency (Cronbach's Alphas equal to 0.91).

### 2.3 Experimental design and procedures

The study was conducted in outdoor settings nested in the neighborhoods where the older adults routinely engage in outdoor activities at different times of the days (see Supplementary material for details of the study sites). Three hundred and nine older adult participants were recruited using convenient sampling across different residential neighborhoods in Singapore and all of the participants were part of our final sample (i.e., no participant was excluded). This allowed us to collect observations from participants with diverse demographic, socio-economic and lifestyle characteristics. Sample size was determined before any data analysis (80% power and alpha error 0.05). The study was conducted at different times of the day, including morning, afternoon, and evening. This ensured that we could capture a range of differing climatic conditions and levels of outdoor thermal comfort. The types of outdoor activities, in which older adults frequently engaged, can be classified into two categories: (1) sedentary activities, involving for example playing chess, chit-chatting, reading newspaper, people or bird watching and (2) physical activities, involving for example Taiji, morning exercises like stretching, running or gardening.

On the day of the experiment, all participants took up their routine outdoor activity for this day (of the week), with the exception that the researchers administered a Stroop task and short questionnaire before the activity and a second Stroop task after they completed their activity. The outdoor activities ranged from 40 to 60 min in duration. The results from the pre-activity Stroop task served as a baseline while results from the post-activity could partially account for the exposure to environmental conditions as older adults engage in their routine activities. Both pre-activity and post-activity Stroop tasks were performed for three consecutive minutes. Before starting the pre-activity Stroop task, participants had a practice session to familiarize themselves with the task and to seek any clarification if necessary. We programmed the Stroop task in-house and administered it using electronic tablets (see Supplementary material for screenshots of the Stroop game).

Once the Stroop tests were completed, a survey questionnaire with three sections was administered to our participants (see Supplementary material for the survey questionnaire). The first section collected sociodemographic information (e.g., gender, age and educational attainment). In the second section, even though our participants were older healthy adults, we asked them to self-rate their general health on a 5-point Likert scale, with 1 being poor and 5 being excellent. This measure was first developed by Ware and Sherbourne (1992) and is now widely used in population health studies.

Besides, we included an additional question to measure for outdoor preference (i.e., amount of time they spent outdoors). A 6-item block of statements were implemented (e.g., it is pleasant to spend time outdoors in Singapore) on a 5-point Likert scale with responses ranging from “strongly disagree” (1) to “strongly agree” (5). Three items focused on spending time outdoors during the day and three items focused on spending time outdoors at night. The mean score was then computed and can range from 1 to 5, with 1 being the least preference of spending time outdoors and 5 being the greatest preference of spending time outdoors. The scales used to measure personal preference toward spending time outdoors demonstrated good internal consistency (Cronbach's Alphas equal to 0.89).

The third section included questions to measure our main variables for analysis, as shown in Figure 1 and Section 2.2. These variables include “attitudes toward the environment”, which indicates pro-environmental attitudes, and “awareness of an environmental problem”, which measures the level of climate change awareness regarding Singapore's climatic conditions and rising urban heat. Lastly, participants provided information about their level of air-conditioning usage at home as a measure for pro-environmental behavior.

### 2.4 Statistical analyses

Analyses were performed using the statistics software STATA 16. To address our research questions and test our hypothesis, we first implemented two linear regressions with the cognitive control capacity as the dependent variable and mean PET and WBGT indexes as predictors. With these regressions, we wanted to explore the relationship between cognitive control capacity and thermal comfort following our framework displayed in Figure 1. To potentially increase the fitness of the regressions, we controlled for socio-demographics (i.e., age, gender, education level and self-rated health) and lifestyle characteristics (i.e., type of activities routinely engaged in -sedentary or physical- and preference for spending time outdoors) of our sample.

Second, and following again Figure 1, we tested whether cognitive control capacity is a moderator between “attitudes toward the environment” and “awareness of the environmental problem” and the “pro-environmental behaviors”. We performed linear regressions in which pro-environmental behavior was introduced as the dependent variable and attitudes and awareness score, and the interaction of these and cognitive control capacity thereof as predictors. To test the specific relevance of the interaction between cognitive control capacity and environmental attitudes and awareness, we calculated a hierarchical linear regression analysis to compare the full model with a model that only contained attitudes and awareness and cognitive control capacity as predictors, but not the interaction. To analyse whether the experience-sampling items measured one underlying construct, we calculated an exploratory factor analysis. In these regressions, we also included demographic, socio-economic and lifestyle characteristics as controls. In this study, we report all measures, manipulations and exclusions.

### 2.5 Sample description

**Figure 2:**
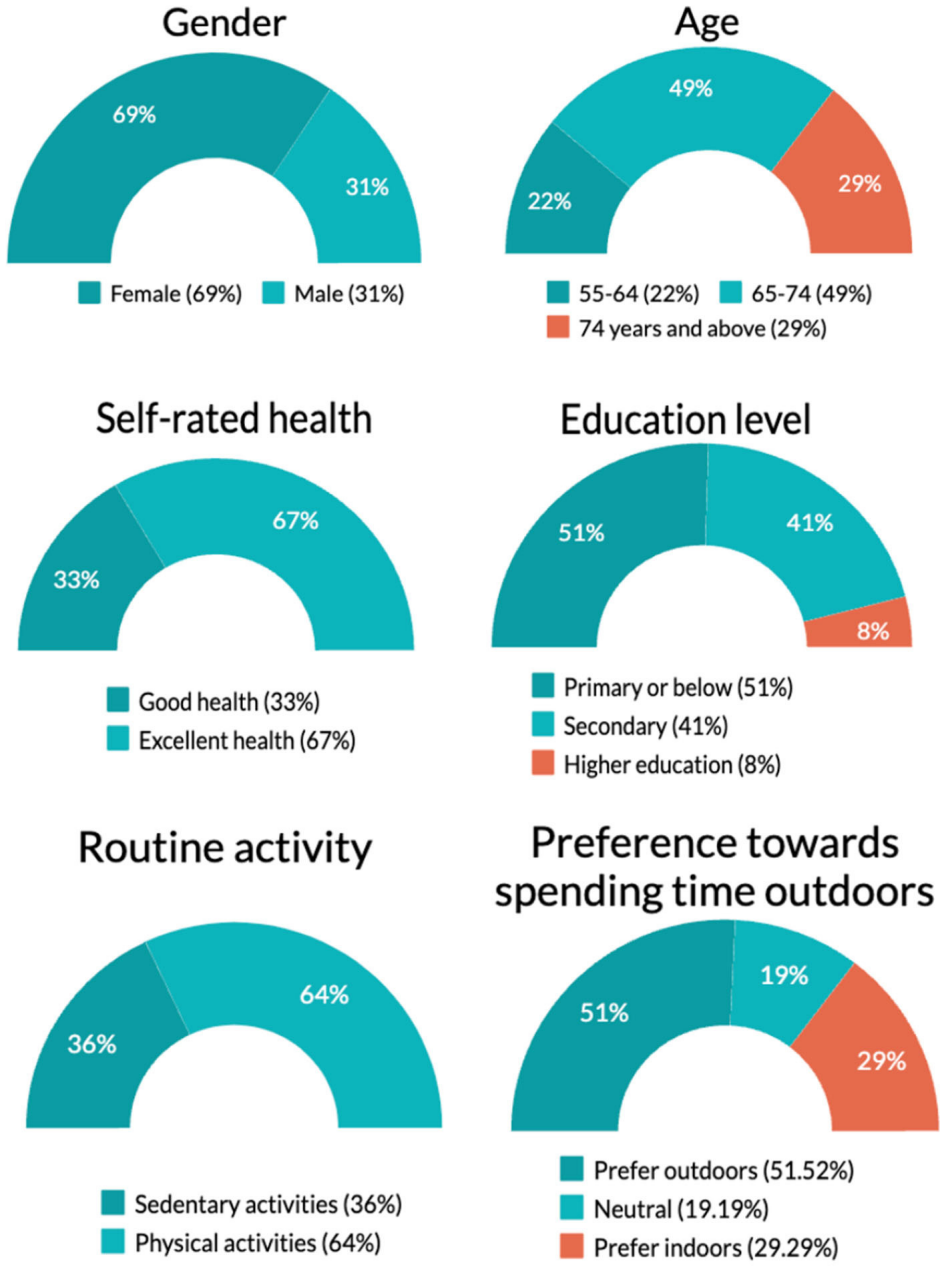
Summary of sample's characteristics (*n* = 309).

Figure 2 shows the sociodemographic and lifestyle characteristics of our sample. Twenty-two precentage of the participants were between 55 and 64 years old, 49% were between 65 and 74 years old and 29% were over 74 years old. Overall, 92% of the sample accomplished at least a primary school education. Sixty-nine percentage of the participants were women. More than two thirds of the participants were positive about their health status. With respect to lifestyles, 64% of our participants routinely are involved in physical activities, while the remaining 36% prefers sedentary activities. Furthermore, 28% of our participants prefer to spend time indoors, 19% are indifferent or neutral while 52% prefer to spend time outdoors.

## 3 Results

The mean pro-environmental behavior score of our entire sample with 309 participants is 3.04 (SE 0.25; range = 1–5). The mean cognitive control score of our sample is 0.38 (SE = 0.04; 95% CI = −0.128 to 1.14). The mean positive pro-environmental attitudes score of our participants is 3.92 (SE = 0.0113; 95% CI = 1–5), while the mean awareness is 3.06 (SE = 0.05; 95% CI = 1–5).

The central hypotheses described in our framework (Figure 1) were tested through econometric analysis. Following it, the first step is to assess the relationship between thermal comfort and cognitive control capacity. To do so, we calculated in two separate linear (i.e., OLS) regressions (Table 1, Column 1 and Column 2) with the average PET and WBGT of each participant during the Stroop tasks to predict their cognitive control capacity. Table 1 Column 1 and Column 2 display the results from the OLS estimations on the cognitive capacity using “Mean PET index” or “Mean WBGT index” as key explanatory variables, respectively.

**Table 1:** OLS regressions for the cognitive control capacity.

**Variables**	**(1)**	**(2)**
	**Cognitive control**	**Cognitive control**
Mean PET index	−0.0500^**^	
	(0.00210)	
Mean WBGT index		−0.0224^***^
		(0.00750)
Education	0.0113	0.00497
	(0.00623)	(0.00625)
Age	−0.00751^***^	−0.00786^***^
	(0.000787)	(0.000851)
Gender (1 = female)	0.0248	0.0214
	(0.0113)	(0.0175)
Preference toward spending time outdoors	0.0211^***^	0.0173^***^
	(0.004)	(0.0050)
Self-rated health status	0.0249^***^	0.0215^***^
	(0.00713)	(0.00708)
Sedentary activities (1 = yes; otherwise = 0)	−0.0887^***^	−0.0961^***^
	(0.0134)	(0.0148)
Constant	0.254^**^	0.164^***^
	(0.106)	(0.0212)
Observations	309	309
*R*-squared	0.133	0.169

Standard errors in parentheses ^***^p < 0.01, ^**^p < 0.05, ^*^p < 0.1.

PET, Physiological Equivalent Temperature; WBGT, Wet Bulb Globe Temperature.

In all regressions, we included demographic and socio-economic characteristics (i.e., education, age, gender and self-perceived health status) as well as lifestyle characteristics of our participants' (i.e., personal preference toward spending time outdoors and the type of activity they routinely engage in) as control variables. But before doing so, we tested for multi-collinearities. As the sociodemographic and lifestyle characteristics were weakly correlated [i.e., coefficients of Pearson's correlation were lower than 0.5 and highly significant (*p* < 0.05); see Supplementary material for full results], they were included as controls variables in our econometric analysis.

Our results from Column 1 and Column 2 in Table 1 show that the coefficients for the “Mean PET index” and “WBGT index” are negative and significant. This indicates that the higher the PET index or the WBGT index, respectively, the poorer the cognitive control capacity is among the participants. This result suggests that improving the outdoor thermal comfort has a positive effect on the cognitive capacity of older adults over 55 years old.

In Table 1 we also see that the coefficients for “Self-rated health status” and “Preference toward spending time outdoors” are positive and significant in all three regressions, suggesting that a higher self-rated health status and preference for spending time outdoors imply a better cognitive control capacity. We also see that the coefficients for “Age” and “Sedentary activities” are negative and significant, suggesting that the cognitive control capacity decreases with age, with the preference for sedentary activities (as opposed to physical activities).

*Result 1: Degrading outdoor thermal comfort conditions affects cognitive control capacities in a negative way for older adults. Cognitive control capacities also tend to decrease with age, preference for sedentary activities and spending time outdoors*.

So far, we have assessed our first hypothesis and provided evidence that thermal comfort affects cognitive control capacity. Now, and following our framework described in Figure 1, we want to assess the role of cognitive control capacity as a mediator of the relationships between attitudes toward the environment and awareness of the environmental problem on pro-environmental behaviors. Table 2 reports the results from the OLS estimations of “Pro-environmental behaviors” using “Attitudes toward the environment”, “Awareness of the environmental problem”, and “Cognitive control capacity” as key explanatory variables. Socio- demographic and lifestyle characteristics were included as control variables.

**Table 2:** OLS estimations for the impact of positive attitudes toward the environment or awareness of environmental problem on mean pro-environmental behaviors taking cognitive control capacities into account.

**Variables**	**(1)**	**(2)**	**(3)**	**(4)**	**(5)**	**(6)**
	**Pro-environmental behaviors**	**Pro-environmental behaviors**	**Pro-environmental behaviors**	**Pro-environmental behaviors**	**Pro-environmental behaviors**	**Pro-environmental behaviors**
Attitudes toward the environment	0.280^**^		0.139^**^		0.292^**^	0.162^**^
	(0.0464)		(0.0357)		(0.0476)	(0.035)
Awareness of environmental problem		0.120^**^		0.159^**^		0.485^**^
		(0.107)		(0.0805)		(0.068)
Cognitive control	1.426	2.562	1.823	2.294	1.356	2.341
	(0.222)	(0.432)	(0.302)	(0.592)	(0.142)	(0.322)
Attitudes toward the environment × cognitive control	0.365^***^		0.373^***^			
	(0.0994)		(0.0763)			
Awareness of environmental problem × cognitive control		1.734^***^		1.935^***^		
		(0.264)		(0.192)		
Education			0.290^***^	0.328^***^		0.324^***^
			(0.0237)	(0.0224)		(0.030)
Age			−0.0184^***^	−0.0176^***^		−0.040^***^
			(0.00295)	(0.00282)		(0.0038)
Gender (1 = female)			0.517^***^	0.394^***^		0.478^***^
			(0.0413)	(0.0402)		(0.0614)
Preference toward spending time outdoors			0.536^***^	0.595^***^		0.453^***^
			(0.0552)	(0.0540)		(0.0418)
Self-rated health status			0.0551	0.0394		0.1081
			(0.0276)	(0.0263)		(0.0337)
Sedentary activities (1 = yes; otherwise = 0)			−0.760^***^	−0.883^***^		−0.720^***^
			(0.0623)	(0.0625)		(0.0621)
Constant	3.726^***^	4.158^***^	4.135^***^	4.158^***^	3.726^***^	4.650^***^
	(0.185)	(0.348)	(0.311)	(0.348)	(0.185)	(0.351)
Observations	309	309	309	309	309	309
*R*-squared	0.098	0.109	0.258	0.255	0.091	0.260

The variables for the Attitudes, Awareness and Cognitive control are mean-centered. Note that in a regression model with an interaction, the other predictors' estimates are only valid for the case that the interaction is zero. Thus, the effect of “Attitude toward the environment” and “Awareness of environmental problem” are only valid if “Cognitive control” is zero (and vice versa). The important aspect of this model, however, is the significant interaction between the awareness and cognitive control as well as between attitudes and cognitive capacity. Standard errors in parentheses; ^***^p < 0.01, ^**^p < 0.05, ^*^p < 0.1.

Column 1 in Table 2 shows the effect of the interaction of attitudes toward urban heat mitigation and an increasing cognitive control capacity. The coefficient for “Attitudes toward the environment × Cognitive control” is positive and highly significant, suggesting that the interaction between these two variables predicts our participants' pro-environmental behavior (*p* = 0.002). The coefficient is statistically significant, explaining 9.8% of the variance in pro-environmental behaviors. The respective R-square value increased by 25.8% when incorporating the control variables into the model (see Column 3). This suggests that socio-demographic and lifestyle characteristics of the participants increased the fit of the model. However, it should be noted that even after adding these control variables, the interaction between attitudes toward urban heat mitigation and cognitive control remained a significant predictor of pro-environmental behavior (*p* = 0.005).

In Column 2 of Table 2, we map the interaction effect of climate change awareness and cognitive control capacities on pro-environmental behaviors. We see that the interaction variable “Awareness of the environmental problem × Cognitive control” is positive and highly significant, suggesting that the interaction between these two variables has a positive effect on the participants' pro-environmental behaviors (*p* = 0.000). In Column 4 of Table 2, we computed the same model but with the addition of the before mentioned control variables. The addition of the control variables increased the fitness of the model, raising the R-square from 10.9% to 22.5%. Again, the interaction terms are still significant (*p* = 0.000). Pearson's correlation tests to check whether “attitudes toward the environment”, “cognitive control capacity” as well as between “awareness of the environmental problem” and “Cognitive control capacity” correlate significantly. This is not the case (*r* = 0.0312; *p* = 0.153 and *r* = 0.0234; *p* = 0.152, respectively).

*Result 2: Cognitive control capacity is a moderator between pro-environmental attitudes and awareness of an environmental problem on the one hand and pro-environmental behaviors on the other hand. The higher the cognitive control capacities, the closer the gap between attitudes and awareness on the one hand and pro-environmental behaviors on the other hand*.

Giving a closer look to the control variables, we observe that “Education” and “Personal preference toward spending time outdoors” play a positive role on pro-environmental behavior, as the coefficients for these variables are positive and significant in Columns (3) and (4) of Table 2. “Female” participants are characterized by more pro-environmental behaviors than male participants. “Age” is significantly and negatively related to pro-environmental behaviors, which means that the older the participants, the less environmentally friendly they behave. For robustness check, we present the analyses without the interaction terms in Columns 5 and 6.

To test the validity of Result 2, we divided the cognitive control capacity into three groups: participants who fall under the 33rd percentile are assigned to the “low” level sub-group; those between the 33rd and 66th percentile to the “medium” level sub-group and those above the 66th percentile are assigned to the “high” level sub-group. We then estimate the interaction terms between the variables “Attitude toward the environment” and “awareness of the environmental problem”, and each of the cognitive control levels (i.e., low, medium and high). Our result confirms that the relationship between pro-environmental attitudes or awareness of the environmental problem and pro-environmental behaviors gets stronger with increasing cognitive control capacities of the participants (see Supplementary material for full results).

## 4 Discussion and recommendations

This study investigates into the role of thermal comfort and cognitive control capacities on pro-environmental behaviors. We do so by first providing evidence that low thermal comfort affects cognitive control, which at the same time is proved to be a significant moderator between pro-environmental attitudes and awareness and pro-environmental behaviors. In fact, we find that the level of cognitive control capacities moderates and strengthens the above-mentioned relationships helping to close both the *attitudes-behavior gap* and the *awareness-behavior gap*.

Our findings extend the results from Langenbach et al. (2020), who provided first evidence that cognitive control capacities may moderate the relationship between pro-environmental attitudes, environmental awareness and pro-environmental behaviors. In addition, it contributes to the emerging body of research on the role of cognitive resources in the study of the pro-environmental attitude-behavior gaps and awareness-behavior gaps. It is also important to highlight the role of socio-demographic and lifestyle characteristics of our sample as significant determinants of better cognitive control capacities and pro-environmental behaviors. Higher levels of education, being female and having personal preferences for spending time outdoors are positive and significant determinants of cognitive control capacity and pro-environmental behaviors, while increasing age and a preference for sedentary activities are negative determinants.

In spite of interesting and relevant insights from our analysis, some limitations must be acknowledged. Our study focused on the pro-environmental behavior of reducing air conditioning use, but it remains unknown if our results would also apply to other pro-environmental behaviors, especially those that are not directly related to heat or humidity conditions (e.g., plastic bag use, recycling or food waste). Another caveat is that we used self-report air conditioning usage, as well as self-reported pro-environmental attitudes and awareness. The reliance on self-reported data introduces potential biases, particularly social desirability and recall bias. Objective measures of air conditioning use would provide a more robust validation of our results.

Our study focused on older adults and thus the results may be different for other age groups of countries' populations, raising questions about whether similar cognitive mechanisms apply across different age groups. Our study was conducted in naturalistic outdoor settings, in which older adults typically engage in outdoor activities. This naturalistic outdoor setting, while enhancing ecological validity, limits the control over external factors such as environmental variability. Further studies offering a higher degree of control of the climatic conditions might be interesting.

Therefore, future research should investigate experimental designs with more controlled climatic conditions, objective behavioral tracking, and a broader range of population samples to strengthen the robustness and generalizability of our findings. Moreover, while our study emphasizes the crucial role of cognitive control capacities in linking thermal comfort to pro-environmental behaviors, future research should examine additional influencing factors, such as emotions and socio-cultural dynamics. Examining how these elements interact could offer deeper insights into overcoming the attitude-behavior and awareness-behavior gaps, ultimately informing more effective strategies for promoting sustainable actions.

The above-mentioned limitations notwithstanding, our study presents evidence that outdoor thermal conditions impact the cognitive control capacities of older adults, which—via the effects of pro-environmental attitudes and climate change awareness on pro-environmental behaviors—is relevant if behavioral changes are aimed at. Improving outdoor thermal comfort would strengthen cognitive control capacities of older adults and could enhance their pro-environmental behaviors. While mitigation measures could be implemented in cities to decrease the impacts of heat, it would be as important to promote individual heat adaptation and acclimatization in tropical countries. In this sense, initiatives that encourage older adults to become more physically active and spend more time outdoors could increase their heat resilience and wellbeing (Rodriguez and D'Alessandro, 2019) while strengthening their cognitive control and fostering sustainable actions like using less air conditioning.

Our findings are particularly timely and significant in light of the global aging population, including in tropical regions. With life expectancy rising, the number of older adults is set to increase dramatically, amplifying the importance of our research. Understanding that better outdoor thermal conditions may improve cognitive control capacities in older adults, while also bridging the pro-environmental attitudes-behavior gap and awareness-behavior gap, contributes to shape more sustainable policies. Cities and their initiatives to promote a more environmentally sustainable and carbon-neutral lifestyle would profit from such knowledge: improving thermal comfort conditions and promoting individual heat adaptation measures would foster the success of their efforts.

## Data Availability

The data are not publicly available due to privacy and ethical restrictions. The anonymised data that support the findings of this study are available on request from the corresponding author.
