# Supplementary material for: Outdoor thermal comfort and cognition impact pro-environmental behaviors: evidence from a field experiment in the tropics
Source: Front Psychol. 2025 May 5;16:1472852. doi: 10.3389/fpsyg.2025.1472852 (PMC12087182; doi:10.3389/fpsyg.2025.1472852)
Supplement: Supplementary file 1 [file Supplementary_file_1.docx]

**Outdoor thermal comfort and cognition impact pro-environmental behaviours: Evidence from a field experiment in the tropics**

***Supplementary material***

1. **Survey Questionnaire**

****Eligibility Check – to be completed by interviewer**

Only Singapore residents or Permanent Residents aged 55 years and above who resides in Punggol can participate in this study.

EC1. Are you a Singapore Citizen or Permanent Resident?

| Yes – Singapore Citizen | Continue with study |
| --- | --- |
| Yes – Permanent Resident |  |
| No | Terminate the study |

EC2. Do you reside in Punggol/Pinnacles/Chinatown?

| Yes | Continue with study |
| --- | --- |
| No |  |

EC3. Please indicate your residential area below: (the first 3 digits of your postal code)

| _____________ (first 3 digits) | Continue with study |
| --- | --- |

EC4. Do you experience any difficulties in identifying different colours?

| Yes | Continue with study |
| --- | --- |
| No | Terminate the study |

EC5. Please identify the numbers you see below:


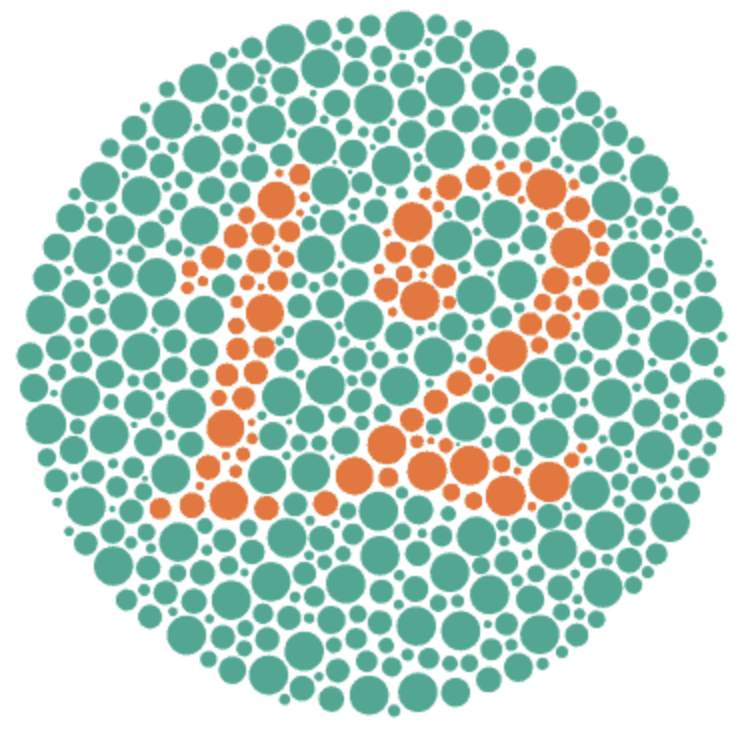

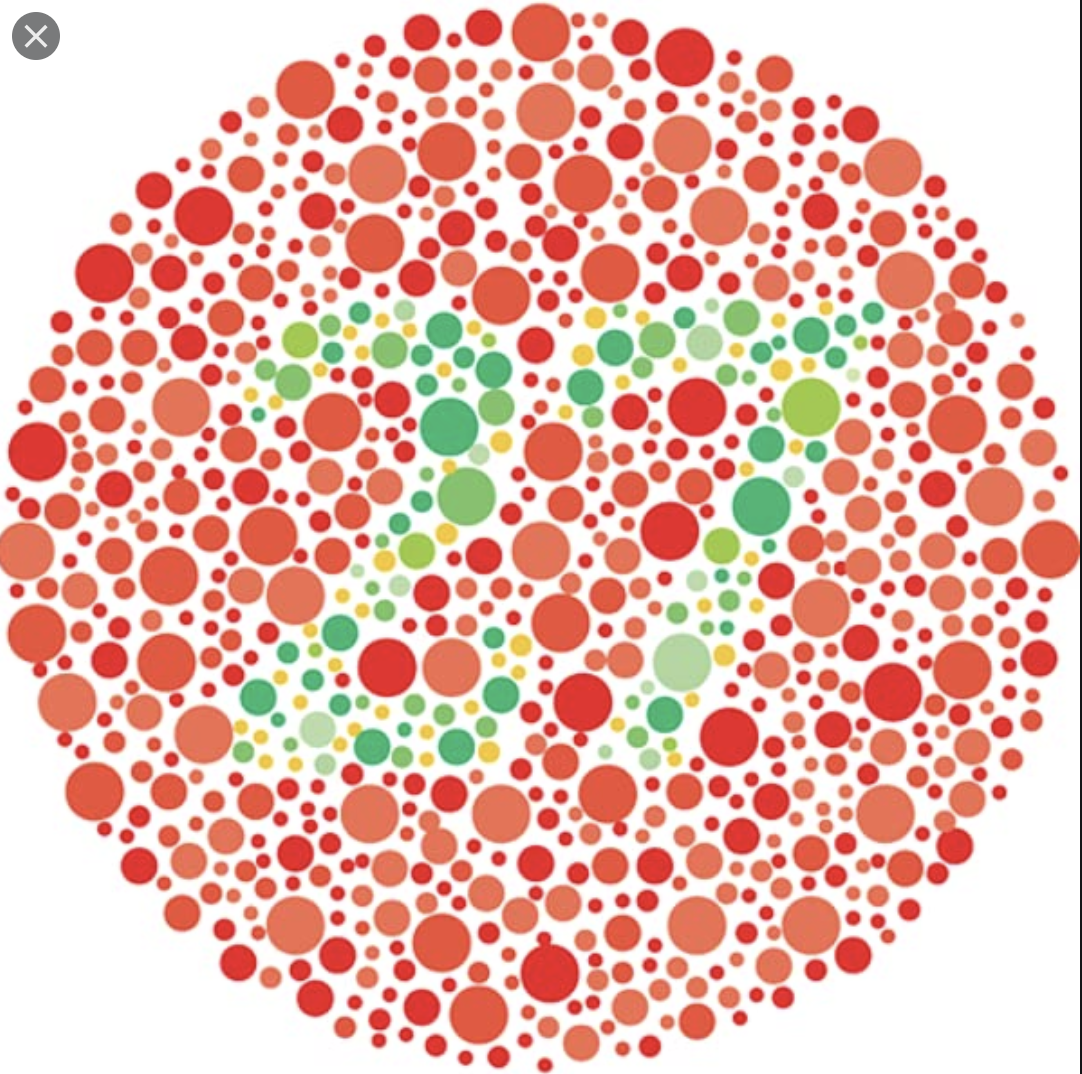

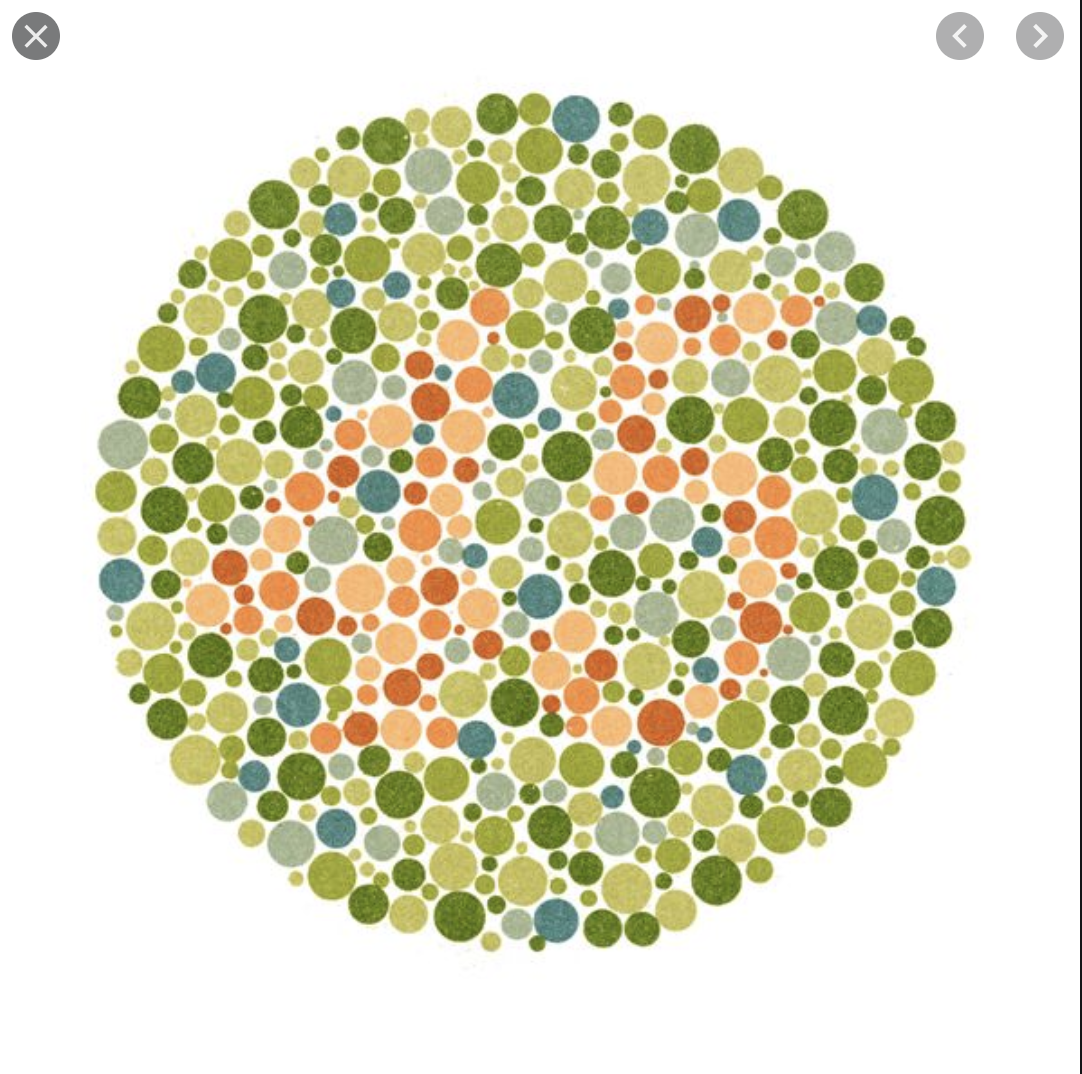


1. Which sex do you identify with?

|  | Male | “Continue” to next question |
| --- | --- | --- |
|  | Female |  |
|  | Prefer not to say |  |

1. What is your ethnicity?

|  | Chinese | “Continue” to next question |
| --- | --- | --- |
|  | Malay |  |
|  | Indian |  |
|  | Others |  |

1. Which year were you born in?

“Continue” to next question

1. What is the highest educational qualifications you have attained?

|  | Primary & below | “Continue” to next question |
| --- | --- | --- |
|  | Secondary |  |
|  | Nitec/Higher Nitec |  |
|  | A Levels / Diploma |  |
|  | Bachelors |  |
|  | Postgraduate |  |
|  | Others  (please specify: _________) |  |
|  | Prefer not to say |  |

1. Over the past 12 months, what is the estimated the average earnings (SGD) of the household per month?

|  | Below $2,000 per month | “Continue” to next question |
| --- | --- | --- |
|  | Between $2,000 to $3,999 |  |
|  | Between $4,000 to $5,999 |  |
|  | Between $6,000 to $9,999 |  |
|  | $10,000 and above |  |
|  | Don’t know |  |
|  | Prefer not to say |  |

1. What type of dwelling do you live in?

|  | HDB 1-Room | “Continue” to next question |
| --- | --- | --- |
|  | HDB 2-Room |  |
|  | HDB 3-Room |  |
|  | HDB 4-Room |  |
|  | HDB 5-Room or Executive Flat |  |
|  | Condominium or Private Flat |  |
|  | Landed Property |  |
|  | Prefer not to say |  |

1. Who are you currently living with?

|  | Alone | “Continue” to next question |
| --- | --- | --- |
|  | Lives with spouse only |  |
|  | Lives with spouse and children |  |
|  | Lives with children only |  |
|  | Lives with children and grandchildren |  |
|  | Lives with other relatives |  |
|  | Lives with friend |  |
|  | Others (please specify: _________) |  |
|  | Prefer not to say |  |

1. How would you rate your health?

|  | Excellent | “Continue” to next question |
| --- | --- | --- |
|  | Very good |  |
|  | Good |  |
|  | Prefer not to say |  |

1. How tall are you (in cm)?

“Continue” to next question

1. How much do you weigh (in kg (in kg)?

“Continue” to next question

1. Do you spend time outdoors?

|  | Yes | “Continue” to next question |
| --- | --- | --- |
|  | No |  |

1. How much time do you spend outdoors each day?

|  | None | “Continue” to next question |
| --- | --- | --- |
|  | 10 to 15 minutes |  |
|  | 30 minutes to 45 minutes |  |
|  | An hour |  |
|  | More than 1 hour but less than 2 hours |  |
|  | More than 2 hours but less than 3 hours |  |
|  | More than 3 hours but less than 6 hours |  |
|  | More than 6 hours |  |

1. What kind of activities do you engage in? **Please select the top three.**

|  | Vigorous physical fitness (dancing, running, jogging, cycling, etc) | “Continue” to next question |
| --- | --- | --- |
|  | Moderate physical exercise (taiji, qigong, stretching, brisk-walking, etc) |  |
|  | Social interactions (gathering, chit-chatting) |  |
|  | Bird / People-watching |  |
|  | Shopping |  |
|  | Eating |  |
|  | Reading newspapers |  |
|  | Others  (please specify: _________) |  |

1. Which places do you visit within your neighbourhood? **Please select the top three.**

|  | Town centre | “Continue” to next question |
| --- | --- | --- |
|  | Fitness corner / facilities |  |
|  | Hawker centre / wet market |  |
|  | Supermarket (s) |  |
|  | Shopping malls |  |
|  | Green spaces (gardens, pocket parks, nature reserve) |  |
|  | Open spaces (playgrounds, void deck, pavilions, multipurpose court) |  |
|  | Community centre |  |
|  | Medical facilities (polyclinics, hospitals, tcm) |  |
|  | Senior activity centres |  |
|  | Others  (please specify: _________) |  |

1. For today’s activity, did you walk for more than 30 minutes before reaching this place?

|  | Yes | “Continue” to next question |
| --- | --- | --- |
|  | No |  |

1. Do you have air-conditioning installed in your dwelling unit?

|  | Yes | “Continue” to next question |
| --- | --- | --- |
|  | No |  |

1. Do you use the air-conditioning systems that you have at home?

|  | Yes | “Continue” to next question |
| --- | --- | --- |
|  | No |  |
|  | Not applicable |  |

1. How frequently do you use the air-conditioning at home?

|  | None | “Continue” to next question |
| --- | --- | --- |
|  | One to two times per week |  |
|  | Two to three times per week |  |
|  | Fortnightly |  |
|  | Everyday |  |

1. Which part of the day would you turn on the air-conditioning more frequently?

|  | Morning (0600 – 1159) | “Continue” to next question |
| --- | --- | --- |
|  | Afternoon (1200 – 1659) |  |
|  | Evening (1700 – 2059) |  |
|  | At night (2100 – 2359) |  |
|  | Midnight / Wee hours  (0000 – 0559) |  |

**Please indicate the extent to which you agree or disagree with the following statements:**

|  |  | Strongly Disagree | Somewhat Disagree | Neutral | Somewhat Agree | Strongly Agree |
| --- | --- | --- | --- | --- | --- | --- |
|  | Using the air-conditioning is something I do automatically |  |  |  |  |  |
|  | Using the air-conditioning is something I do without having to consciously remember |  |  |  |  |  |
|  | Using the air-conditioning is something I do without thinking |  |  |  |  |  |
|  | Using the air-conditioning is something I start doing before I realize I’m doing it |  |  |  |  |  |
| “Continue” to next question | | | | | | |

**Please indicate the extent to which you agree or disagree with the following statements:**

|  |  | Strongly Disagree | Somewhat Disagree | Neutral | Somewhat Agree | Strongly Agree |
| --- | --- | --- | --- | --- | --- | --- |
|  | In general, I prefer to be outdoors than indoors |  |  |  |  |  |
|  | During the day, I prefer to be outdoors than indoors |  |  |  |  |  |
|  | During the night, I prefer to be outdoors than indoors |  |  |  |  |  |
|  | Spending time outdoors (compared to indoors) is unenjoyable for me |  |  |  |  |  |
|  | Spending time outdoors (compared to indoors) is pleasant for me during the day |  |  |  |  |  |
|  | Spending time outdoors (compared to indoors) is pleasant for me during the night |  |  |  |  |  |
| “Continue” to next question | | | | | | |

**Please indicate the extent to which you agree or disagree with the following statements:**

|  |  | Strongly Disagree | Somewhat Disagree | Neutral | Somewhat Agree | Strongly Agree |
| --- | --- | --- | --- | --- | --- | --- |
|  | If applicable, compared to 5 years ago, Singapore is much warmer now |  |  |  |  |  |
|  | If applicable, compared to 5 years ago, Singapore is much cooler now |  |  |  |  |  |
|  | The changing climate in Singapore is an urgent problem |  |  |  |  |  |
|  | Mitigation action needs to be taken for Singapore’s changing climate |  |  |  |  |  |
|  | More resources should be allotted to address the changes in climatic conditions faced in Singapore |  |  |  |  |  |

**** TO BE FILLED IN BY THE RESEARCHER**

Please report:

**Date** where survey questionnaire is filled:

**Time** where survey questionnaire is filled:

*Please indicate the clothing of the participants:*

-----------------------------------------------------------------------------------------------------------

**Top:** Short sleeves t-shirt Long sleeves t-shirt Suits jacket

-----------------------------------------------------------------------------------------------------------

**Bottom:** Short pants Short skirt Long pants

Long skirt Jeans

-----------------------------------------------------------------------------------------------------------

**Shoes:** Closed-toes shoes Sandals/Flip flops

-----------------------------------------------------------------------------------------------------------

**Others:** Hat Umbrella. Trolley/Bulky bags

None

-----------------------------------------------------------------------------------------------------------

## Definition of environmental variables and thermal indexes

**Climatic Conditions**

1. *Relative humidity*

Relative humidity refers to the percentage of moisture in the air relative to the total amount of moisture the air.

1. *Wind speed*

Wind speed refers to the rate at which air is moving and it is measured in terms of miles per hour (m/h).

1. *Air temperature*

Air temperature refers to the measure of how hot or cold the air is. Using the kestrels, air temperature reading is measured in terms of degree Celcius (°C).

1. *Mean radiant temperature (T_mrt_)*

T_mrt_ summarizes the effects of heat fluxes that reaches the human body (Kantor & Unger, 2011). The globe temperature measurements captured on-site will be first transformed into an outdoor mean radiant temperature that reflects the amount of heat fluxes experienced. The transformed mean radiant temperature (T_mrt_) formula (ISO, 1998) is found below:

$$T_{mrt}=\left[ \left( t_{g}+273 \right)^{4}+\frac{1,1*{10}^{8}*v_{a}^{0.6}}{\varepsilon_{g}*D^{0.4}}(t_{g}-t_{a} \right]^{\frac{1}{4}}-273$$

Where $t_{g}$ = Globe Temperature (°C);

$v$_a_ = Wind Speed at the level of the globe;

$t_{a}$_._ = Air Temperature

Globe temperature assesses a combination of solar radiation, air temperature and wind spend on human comfort. It is measured in terms of degree Celcius (°C).

**Physiological Equivalent Temperature (PET)**

PET is defined as “the equivalent temperature to air temperature in which in a reference environment, the thermal balance and the skin and the core temperatures are the same of that found in the environment” (Hoppe, 1999). Adapted from the Munich Energy-Balance model, PET integrates the complex thermal conditions in an outdoor environment with his/her experiences indoor. The calculation of each participant’s PET includes three parameters: physical constraints, personal variables and climate variables. Physical constraints consist of blood density, blood specific heat, air specific heat, skin emissivity, clothing emissivity and latent heat of evaporation. Personal variables include the age, sex, weight, height, metabolic activity level and the clothing level of the participants. Climatic variables refer to air temperature, air relative humidity, wind speed and T_mrt_.

**Wet bulb globe temperature (WBGT)**

It is a perceived or apparent temperature. It is more a measure of how the weather feels than the exact weather itself. Specifically, WBGT is a measure of stress caused by the heat of direct sunlight. To be accurate, one must take a variety of factors into account; temperature, humidity, wind speed, sun angle, and cloud cover all affect sunlight, heat, and the stress perceived by the body while subject to the conditions in question. Since WBGT is a measure of heat-related stress specifically, it is an important factor to consider anytime people are performing some kind of outdoor activity. When water evaporates into the air it creates a cooling effect, but the amount of this cooling varies depending on the overall air temperature and the relative humidity. The wet-bulb temperature is the lowest possible temperature that air can be cooled down to by the evaporation of water alone, given constant pressure.

This is represented by a rather complex formula (see link):

WBGT = T * arctan[0.151977 * (rh% + 8.313659)^(1/2)] + arctan(T + rh%) – arctan(rh% – 1.676331) + 0.00391838 *(rh%)^(3/2) * arctan(0.023101 * rh%) – 4.686035

Where, “T” is the temperature in Celsius and “rh” represents the relative humidity.

## Description of study neighbourhoods & study sites

The Punggol neighbourhood integrated living, transactional and entertainment services, social amenities and facilities for physical activities with different transportation modes for increased convenience. The integrated living model was further enhanced into tree-lined boulevard as the Punggol Waterway was constructed. Meanwhile, the CBD neighbourhood located within close proximity to the Downtown core and its vicinity is surrounded by numerous tall commercial buildings. Embedded within the neighbourhood were impressive skyscrapers and tourist attractions as fast-paced commercial and financial activities take place within these tall buildings


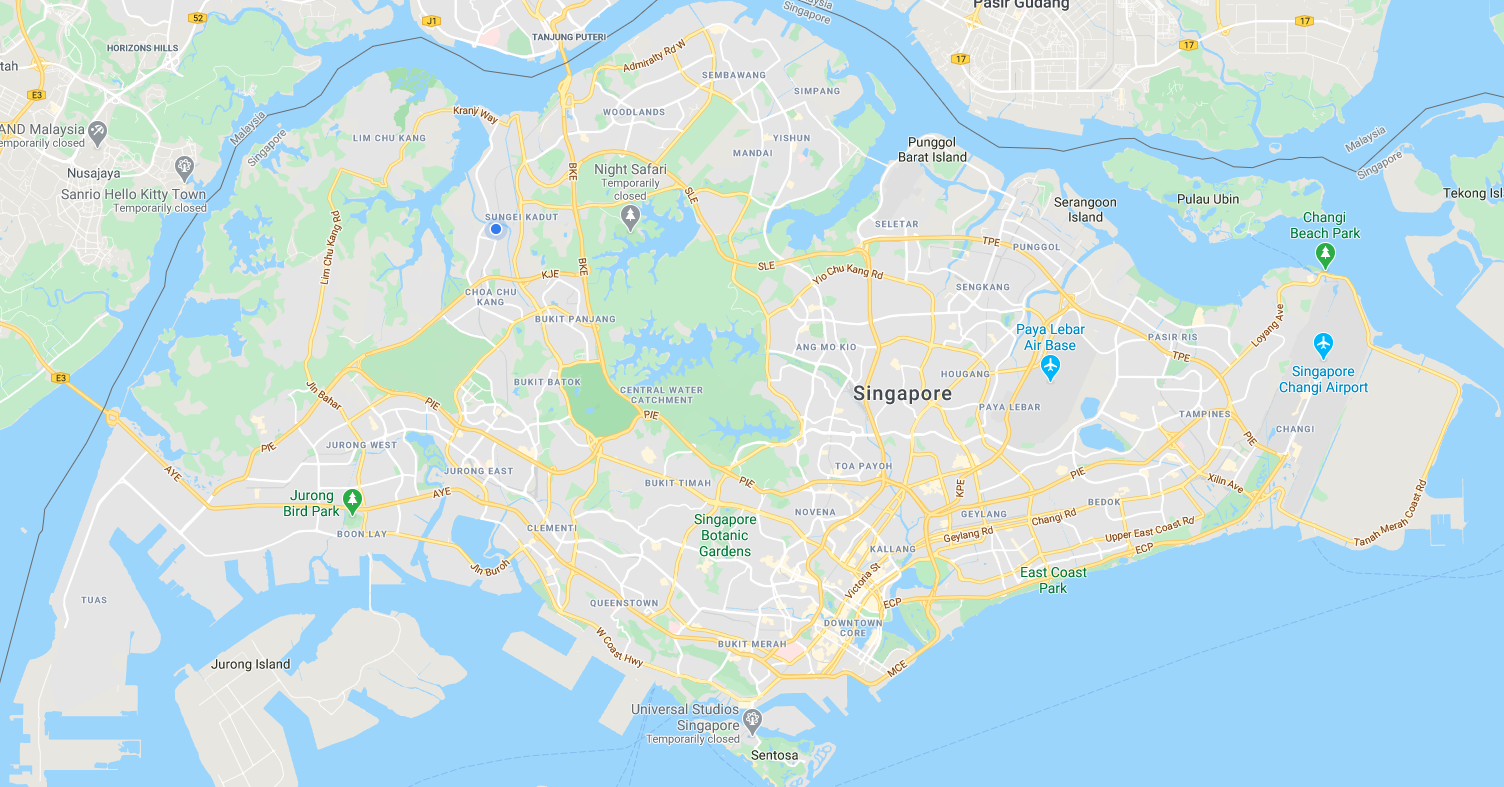


CBD area

Punggol

*Map of identified neighbourhoods*

*Comparison of mean air temperature between neighbourhoods*

Outdoor spaces in both neighbourhoods were identified through site visits using three criteria: (i) the outdoor spaces should be places where older adults congregate for activities, (ii) the outdoor spaces should include residential area with mixed land use, and (iii) the activities conduced in these outdoor spaces should be conducted during similar times of the day and have comparable metabolic equivalent of task ratings (Oke et al., 2017).

It is important to note that the annual mean air temperature (data provided by Meteorological Service Singapore) of the different neighbourhoods between 2010 – 2020 were compared and no significant differences were observed. See graph above.

##
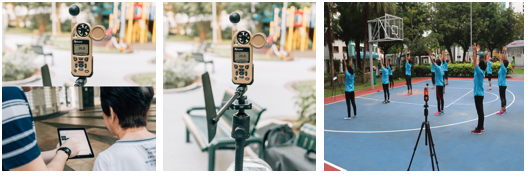
Image of Kestrel 5400 Heat Stress Meters Deployed

***Figure 8****. Photos of tripod-mounted kestrels deployed on-site for data collection*

## Stroop Game Screenshots


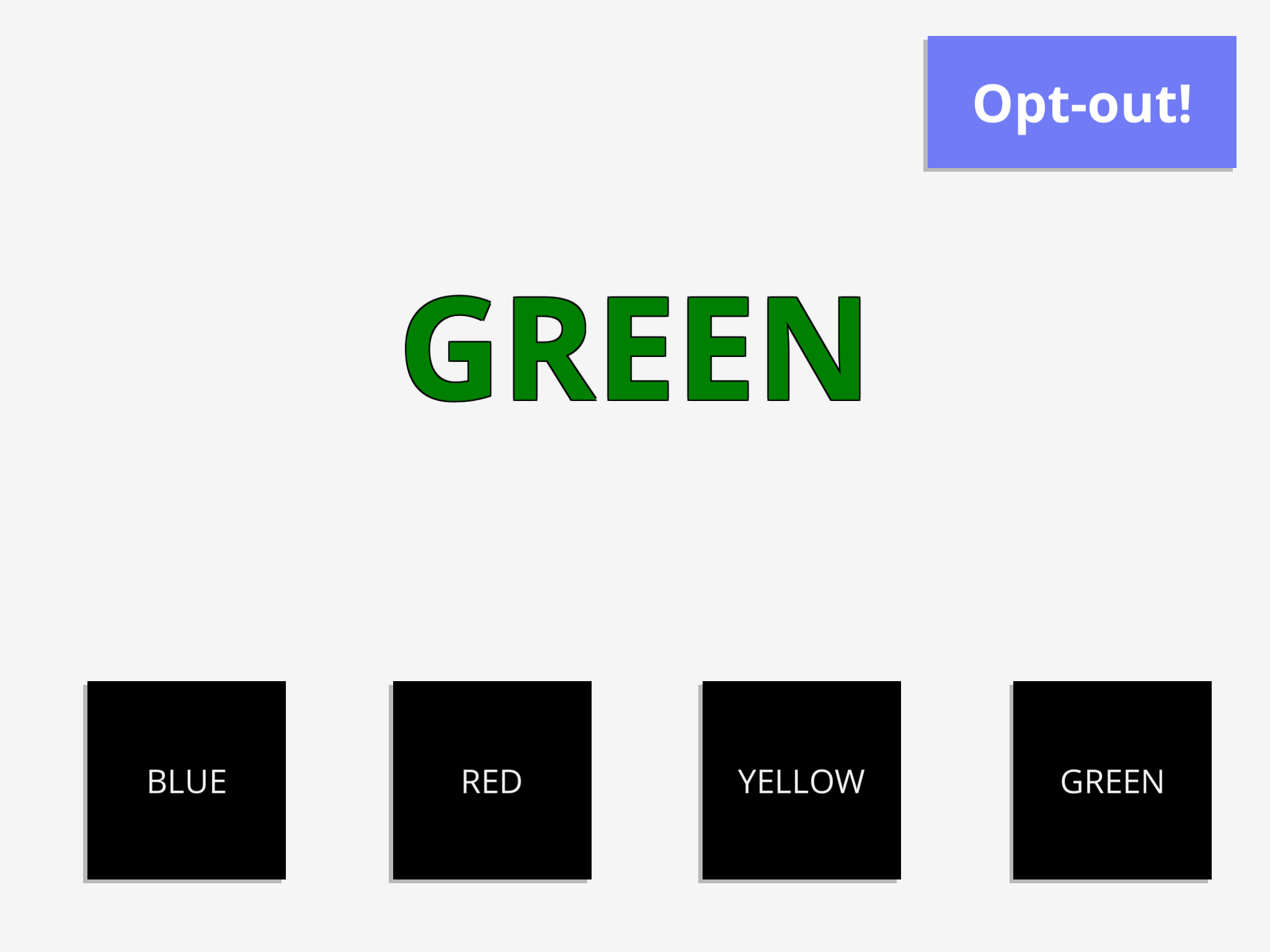

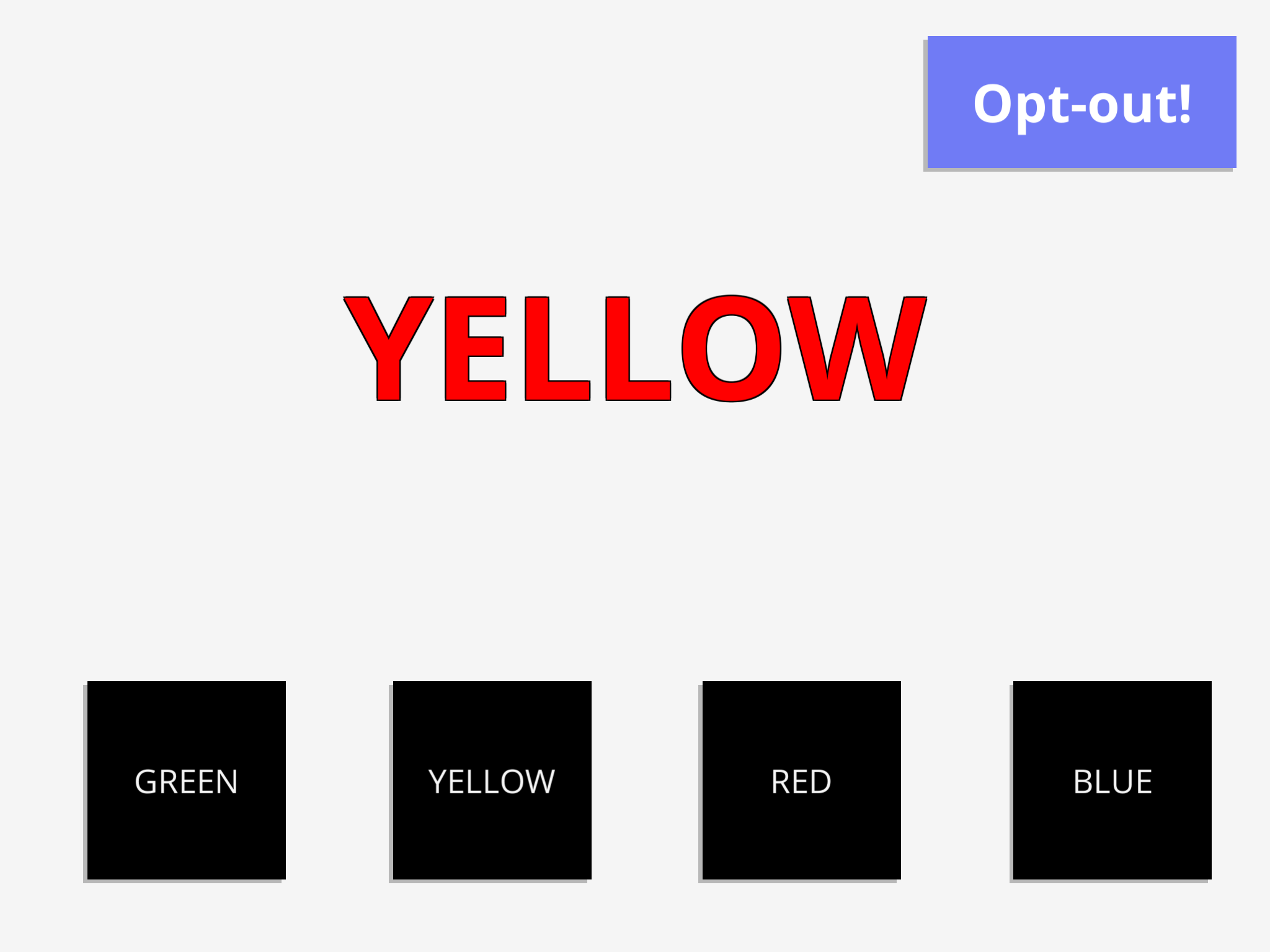


***Figure 6a****. Example of a congruent question* ***Figure 6b.*** *Example of an incongruent question*


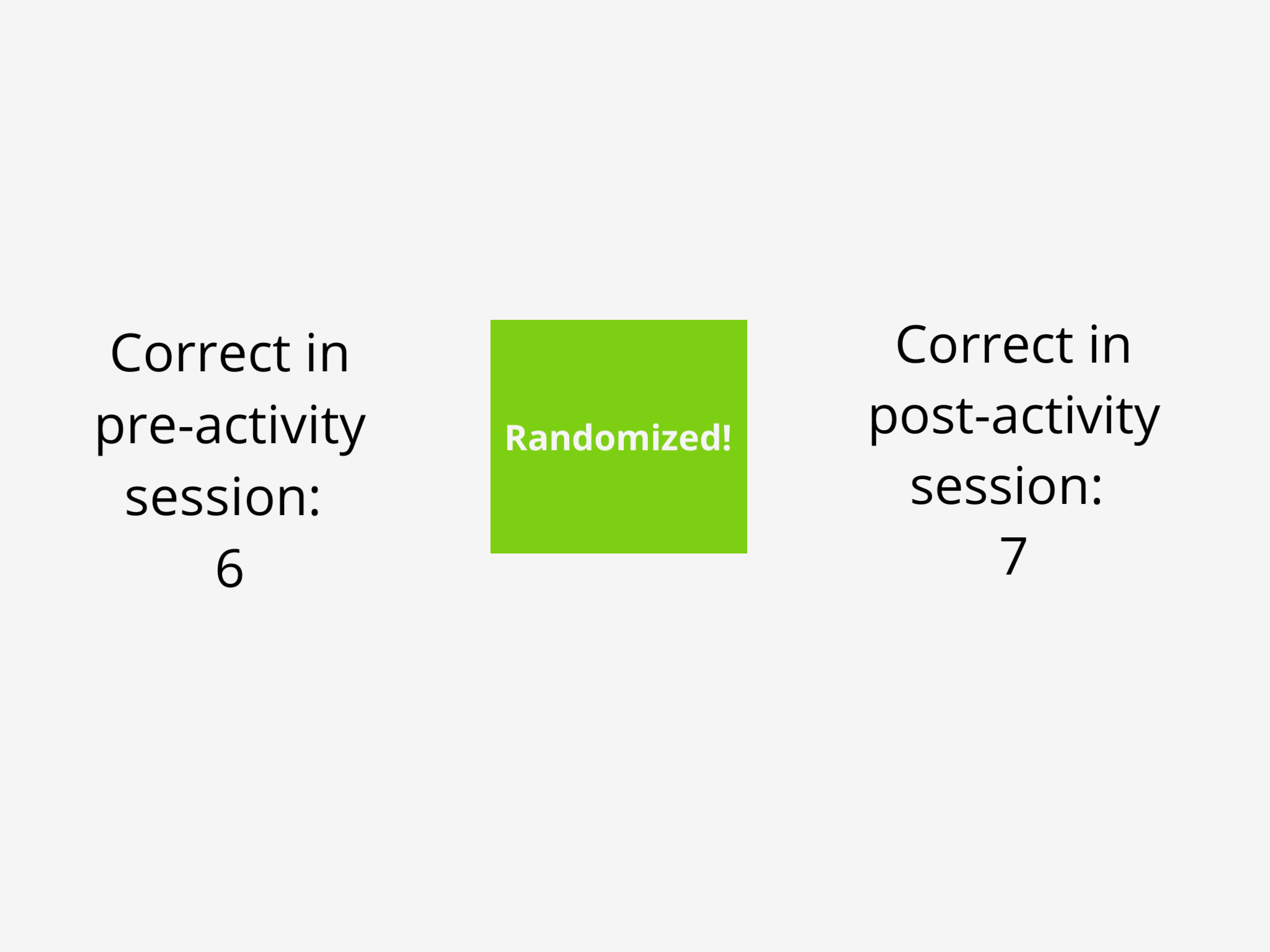


***Figure 7****. Screenshot of scoreboard for both Stroop game session*

1. **Table 1. Pearson’s correlation tests**

|  | Educational Attainment | Age | Gender | Preference towards spending time    outdoors | Self-rated health status | Sedentary activities |
| --- | --- | --- | --- | --- | --- | --- |
| Education | 1 |  |  |  |  |  |
| Age | -0.1812 | 1 |  |  |  |  |
| Gender | 0.0616 | 0.1025 | 1 |  |  |  |
| Preference towards spending time outdoors | 0.1305 | -0.0598 | -0.0606 | 1 |  |  |
| Self-rated health status | 0.2229 | 0.2016 | 0.0216 | 0.0654 | 1 |  |
| Sedentary activities | -0.185 | -0.319 | -0.3259 | -0.0234 | -0.1094 | 1 |

1. **Table 2: OLS estimations for the impact of pro-environmental attitudes or awareness on pro-environmental behaviours taking different levels of cognitive control capacities into account**

|  | (1) | (2) |
| --- | --- | --- |
| VARIABLES | Pro-  environmental behaviour | Pro-environmental behaviour |
| Attitude towards the environment | 0.406** |  |
|  | (-0.0455) |  |
| Awareness of environmental problem | | 1.241** |
|  |  | (-0.0885) |
| Level of cognitive control (1=low, the ref; 2=medium; 3=high) | 0.859 | 1.508 |
|  | (-0.133) | (-0.193) |
| **Attitudes towards the environment x  LOW level of cognitive control** | **Ref** |  |
| **Attitudes towards the environment x  MIDDLE level of cognitive control** | **0.173***** |  |
|  | (-0.0362) |  |
| **Attitudes towards the environment x HIGH level of cognitive control** | **0.354***** |  |
|  | (-0.0675) |  |
| **Awareness of environmental problem x LOW level of cognitive control** | | **Ref** |
| **Awareness of environmental problem x MIDDLE level of cognitive control** | | **0.425***** |
|  |  | (-0.0648) |
| **Awareness of environmental problem x HIGH level of cognitive control** | | **0.888***** |
|  |  | (-0.125) |
| Controls | YES | YES |
|  |  |  |
| Constant | -1.702*** | 1.276** |
|  | (-0.423) | (-0.567) |
| Observations | 309 | 309 |
| R-squared | 0.282 | 0.256 |
| Note: positive attitudes towards heat mitigation, climate change awareness and  cognitive ability level are mean-centred. Note that in a regression model with an interaction, the other predictors’ estimates are only valid for the case that the interaction is zero. Thus, the effect of “Attitudes towards the environment” and “Awareness of environmental problem” are only valid if “cognitive control capacity level” is zero (and vice versa). The important aspect of this model, however, is the significant interaction between the awareness and each cognitive control capacity level in Column 2 as well as between pro-environmental attitudes and  each cognitive control capacity level in Column 1. As in Table 4, we included controls for age, education, personal preference towards spending time outdoors, self-rated health status and type of activity.  Standard errors in parentheses  *** p<0.01, ** p<0.05, * p<0.1. | | |
